# Diversity, Distribution, and Evolution of Bioluminescent Fungi

**DOI:** 10.3390/jof11010019

**Published:** 2024-12-31

**Authors:** Brian A. Perry, Dennis E. Desjardin, Cassius V. Stevani

**Affiliations:** 1 Department of Biological Sciences, California State University East Bay, Hayward, CA 94542, USA; 2 Department of Biology, San Francisco State University, San Francisco, CA 94132, USA; ded@sfsu.edu; 3 Departamento de Química Fundamental, Instituto de Química, Universidade de São Paulo, São Paulo 05508-000, SP, Brazil; 4 Departamento de Bioquímica, Instituto de Química, Universidade de São Paulo, São Paulo 05508-000, SP, Brazil

**Keywords:** Agaricales, basidiomycetes, bioluminescence, caffeic acid cycle, systematics, taxonomy

## Abstract

All known bioluminescent fungi are basidiomycetes belonging to the Agaricales. They emit 520–530 nm wavelength light 24 h per day in a circadian rhythm. The number of known bioluminescent fungi has more than doubled in the past 15 years from 64 to 132 species. We currently recognize five distinct lineages of bioluminescent Agaricales belonging to the Omphalotaceae (18 species), Physalacriaceae (14), Mycenaceae (96), Lucentipes lineage (3), and Cyphellopsidaceae (1). They are distributed across the globe with the highest diversity occurring on woody or leafy substrates in subtropical closed canopy forests with high plant diversity. With the caveat that most regions of the world have not been extensively sampled for bioluminescent fungi, the areas with the most known species are Japan (36), South America (30), North America (27), Malesia, South Asia, and Southeast Asia (26), Europe (23), Central America (21), China (13), Africa (10), Australasia, Papua New Guinea, and New Caledonia (11), and the Pacific Islands (5). Recent studies have elucidated the biochemical and genetic pathways of fungal bioluminescence and suggest the phenomenon originated a single time early in the evolution of the Agaricales. Multiple independent evolutionary losses explain the absence of luminescence in many species found within the five lineages and in the majority of Agaricales.

## 1. Introduction

### 1.1. Reports of Bioluminescent Fungi from BCE Through 2008

Humans have been fascinated with terrestrial bioluminescence for thousands of years, with the earliest documentation dating to Aristotle (384–322 BCE) who described light emission from rotten wood [1]. Sporadic reports of this phenomenon occurred throughout the subsequent centuries until the early nineteenth century when J.F. Heller (1813–1871), professor at Vienna University, was the first to correlate cause and effect attributing light emission from wood to fungi (for a review of these early reports see [2]). Wassink [3] was the first to provide a comprehensive accounting of bioluminescent fungi species, initially reporting 19 species, then expanding the list thirty years later [4], treating 42 taxa with verified or questionable luminescent properties. He also provided a list of 33 species names of uncertain taxonomic position and of doubtful bioluminescent capabilities. 

Desjardin et al. [2] re-evaluated Wassink’s [4] list of luminescent taxa, surveyed the literature post-1978, and coupled with their extensive fieldwork, herbarium research, and molecular analyses provided an updated accounting of bioluminescent fungi. In their review, they recognized 64 species of bioluminescent, mushroom-forming, saprotrophic or rarely plant pathogenic, white-spored euagarics all belonging to the Agaricales (Basidiomycota). Three distinct lineages were reported, annotated as the *Omphalotus* lineage (Omphalotaceae—12 species), *Armillaria* lineage (Physalacriaceae—5 species), and Mycenoid lineage (Mycenaceae—45 species). In addition, they noted that based on unpublished molecular phylogenetic data, two luminescent species, *Gerronema viridilucens* and *Mycena lucentipes*, belonged outside of the other three lineages and may represent a fourth independent lineage [2].

### 1.2. Reports of Bioluminescent Fungi After 2008

In the past fifteen years, many researchers have described new species of bioluminescent mushrooms or reported previously described species newly recognized as emitting light, more than doubling the number known (Table 1). We currently recognize 132 taxa of bioluminescent fungi, all species of Basidiomycota belonging to the Agaricales, representing five distinct lineages: the *Omphalotus* lineage (Omphalotaceae—18 species), *Armillaria* lineage (Physalacriaceae—14 species), Mycenoid lineage (Mycenaceae—96 species), Lucentipes lineage (Cyphellaceae/Porotheleaceae—3 species), and the recently discovered *Eoscyphella* lineage (Cyphellopsidaceae—1 species [5]). An account of these new reports and their global distribution is provided below.

## 2. Taxonomic Review of Bioluminescent Fungal Lineages

### 2.1. Omphalotus Lineage (Omphalotaceae)

**Figure 1:**
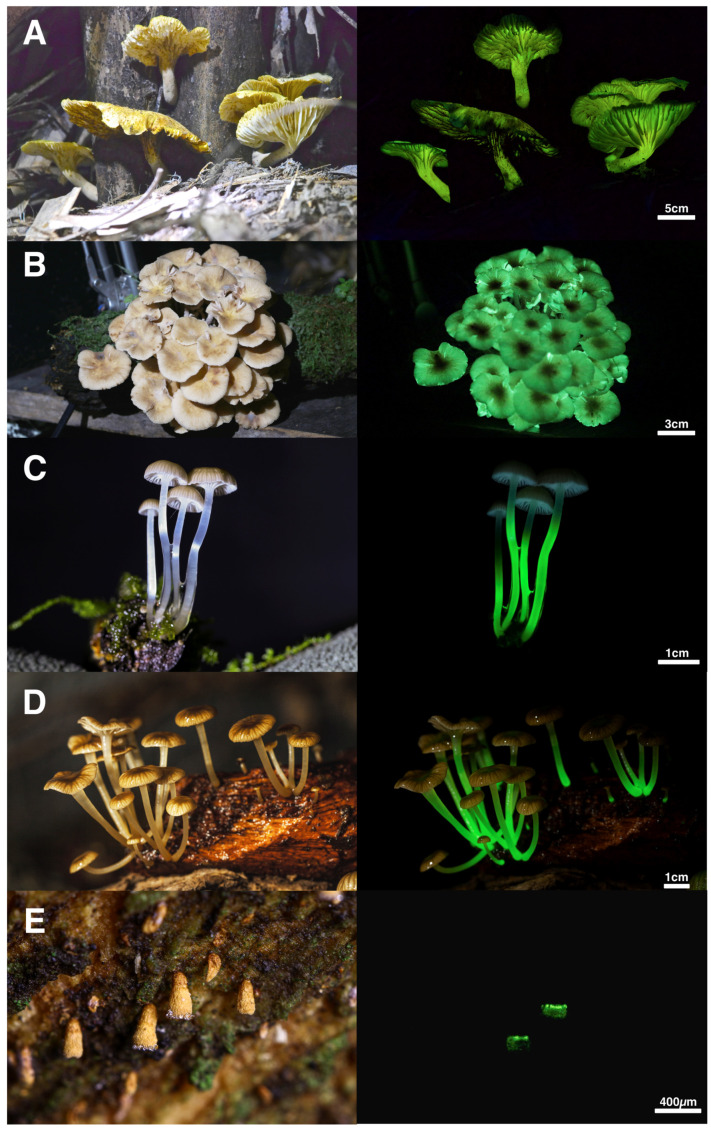
Exemplary bioluminescent mushrooms from the five distinct evolutionary lineages present in Brazil: (**A**) *Neonothopanus gardneri* (*Omphalotus* lineage), (**B**) *Armillaria* sp. (*Armillaria* lineage), (**C**) *Mycena luxaeterna* (Mycenoid lineage), (**D**) *Mycena lucentipes* (Lucentipes lineage), and (**E**) *Eoscyphella luciurceolata* (*Eoscyphella* lineage).

Members of the *Omphalotus* lineage are commonly known as jack-o-lantern mushrooms (*Omphalotus olearius*, *O. illudens*, *O. subilludens*, *O. olivascens*), ghost fungus (*O. nidiformis*), or moon night mushroom (*O. japonicus*), and have been documented since the time of Pliny the Elder. They form large, fleshy mushrooms with decurrent lamellae, are lignicolous saprotrophs of hardwoods, and produce the toxic sesquiterpene Illudin S (lampterol) [2]. Desjardin et al. [2] reported 12 distinct taxa in this lineage, all with luminescent basidiomes. The positive luminescent properties of their mycelium were known at the time from only three species. Since then, four additional species have been found to emit light from their basidiomes, of which three also have a luminescent mycelium. We suspect that all species of *Omphalotus* have luminescent basidiomes and mycelia, and future research should focus on documenting this prediction. Included in the new additions to the list is *Neonothopanus gardneri* (Figure 1A), a long-forgotten species rediscovered in Brazil [6] and used to study the enzymatic nature of bioluminescence in fungi [7,8], to prove that all fungi share the same mechanism for light emission [9], the circadian rhythm of light emission [10], the structure of the luciferin precursor [11], and the chemical and biochemical mechanism of light emission by the Caffeic Acid Cycle (CAC) [12,13]. Only three genera are currently recognized in this lineage, *Omphalotus*, *Nothopanus*, and *Neonothopanus*. The genus *Lampteromyces* is accepted as a synonym of *Omphalotus*, and the four species reported as luminescent *Pleurotus* (Table 1) need further evaluation. We strongly suspect that two species recently described in *Marasmiellus* [14] represent members of the *Omphalotus* lineage (see below).

### 2.2. Armillaria Lineage (Physalacriaceae)

Collectively known as honey mushrooms, *Armillaria* species typically form clusters of relatively large edible basidiomes that are facultative saprotrophs or white rot root pathogens of a variety of trees, shrubs, and woody herbaceous perennial plants (Figure 1B). Species typically form creamy-white mycelial fans and coarse black rhizomorphs in the soil or under the bark of host plants, forming basidiomes seasonally when conditions are appropriate [2]. Individual genets of selected species can be quite large and long-lived. Ferguson et al. [15] reported an individual of *A. ostoyae* that covered 900 hectares (9 km^2^) and was estimated to be between 2000 and 8500 years old.

**Table 1:** Species of fungi reported as bioluminescent in the literature, distributed in five evolutionary lineages.

Taxon (*1)	Mycelium/Basidiome	Distribution (*2)	Citations (*3)
*Omphalotus* lineage—Omphalotaceae (*4)			
1. *Lampteromyces luminescens* M. Zang	?/+	CH	[16,17]
2. *Neonothopanus gardneri* (Berk.) Capelari et al. = *Pleurotus gardneri* (Berk.) Sacc.	+/+	SA	[6,18]
3. *Neonothopanus nambi* (Speg.) Petersen & Krisai-Greilhuber = *Nothopanus eugrammus* (Mont.) Singer *sensu* Corner *non sensu* Singer	+/+	SA, CA, MS, AU	[19,20]
4. *Nothopanus noctilucens* (Lév.) Singer = *Pleurotus noctilucens* Lév.	?/+	JP	[21,22]
5. *Omphalotus illudens* (Schwein.) Bresinsky & Besl. = *Clitocybe illudens* Schwein. = *Panus illudens* (Schwein.) Fr. = *Pleurotus facifer* Berk. & M.A. Curtis	+/+	EU, NA	[3,4,23]
6. *Omphalotus flagelliformis* Zhu L. Yang & B. Feng	?/+	CH	[17]
7. *Omphalotus japonicus* (Kawam.) Kirchm. & O.K. Mill. = *Lampteromyces japonicus* (Kawam.) Singer = *Pleurotus japonicus* Kawam. = *Omphalotus guepiniiformis* (Berk.) Neda	+/+	JP	[17,24,25,26,27,28] Conserved against the older epithet *Agaricus guepiniiformis* Berk. [29]
8. *Omphalotus mangensis* (Jian Z. Li & X.W Hu) Kirchm. & O.K. Mill. = *Lampteromyces mangensis* Jian Z. Li & X.W Hu	?/+	CH	[30,31]
9. *Omphalotus nidiformis* (Berk.) O.K. Mill. = *Pleurotus nidiformis* (Berk.) Sacc. = *Pleurotus candescens* (F. Muell. & Berk.) Sacc. = *Pleurotus illuminans* (Berk.) Sacc. = *Pleurotus lampas* (Berk.) Sacc. = *Pleurotus phosphorus* (Berk.) Sacc.	?/+	AU	[32,33,34]
10. *Omphalotus olearius* (DC.: Fr.) Singer = *Pleurotus olearius* (DC.) Gillet	+/+	EU	[3]
11. *Omphalotus olivascens* H.E. Bigelow, O.K. Mill. & Thiers	+/+	NA	[35] Desjardin (pers. obs.)
12. *Omphalotus subilludens* (Murrill) H.E. Bigelow	?/+	NA	Lockwood (pers. comm.)
13. *Pleurotus decipiens* Corner	?/+	MS	[19]
14. *Pleurotus eugrammus* var. *radicicolus* Corner	?/+	MS, JP	[19]
15. *Pleurotus nitidis* Har. Takah. & Taneyama = *Pleurotus lunaillustris* Kawam. *nom. inval*	?/+	JP	[14]
16. *Pleurotus luminosus* Beeli	?/+	AF	[36]
**Uncertain Placement** described in *Marasmiellus*, but probably belongs in *Omphalotus*			
17. *Marasmiellus lucidus* Har. Takah., Taneyama & S. Kurogi	?/+	JP	[14]
18. *Marasmiellus venosus* Har. Takah., Taneyama & A. Hadano	+/+	JP	[14]
***Armillaria* lineage—Physalacriaceae**			
1. *Armillaria borealis* Marxm. & Korhonen	+/−	EU	[37]
2. *Armillaria calvescens* Bérubé & Dessur.	+/−	NA	[38]
3. *Armillaria cepistipes* Velen.	+/−	NA, JP	[38,39]
4. *Armillaria fuscipes* Petch	+/−	MS	[3,4,23]
5. *Armillaria gallica* Marxm. & Romagn	+/−	EU, NA, JP	[38,39,40]
6. *Armillaria gemina* Bérubé & Dessur.	+/−	NA	[38]
7. *Armillaria limonea* (G. Stev.) Boesew	?/+	AU	[41]
8. *Armillaria mellea* (Valh.) P. Kumm. *sensu stricto * = *Armillariella mellea* (Valh.) P. Karst.	+/−	EU, NA, JP	[38,39,40]
9. *Armillaria nabsnona* T.J Volk & Burds.	+/−	NA, JP	[38,39]
10. *Armillaria ostoyae* (Romagn.) Henrik	+/−	EU, NA, JP	[38,39,42]
11. *Armillaria sinapina* Bérubé & Dessur.	+/−	NA	[38]
12. *Armillaria* sp.	+/+	SA	Desjardin et al. (pers. obs.)
13. *Desarmillaria ectypa* (Fr.) R.A. Koch & Aime = *Armillaria ectypa* (Fr.) Lamoure	+/+	EU	[43]
14. *Desarmillaria tabescens* (Scop.) R.A. Koch & Aime = *Collybia tabescens* (Scop.) Fr. = *Armillaria tabescens* (Scop.) Emel	+/−	EU, NA, JP	[38,40,44]
***Lucentipes* lineage—Cyphellaceae/Porotheleaeceae**			
1. *Gerronema viridilucens* Desjardin, Capelari & Stevani	+/+	SA	[45]
2. *Mycena lucentipes* Desjardin, Capelari & Stevani	+/+	SA, CA	[46]
3. *Mycena quiniaultensis* Kauffman	?/+	NA	[47]
***Eoscyphella* lineage—Cyphellopsidaceae**			
1. *Eoscyphella luciurceolata* Silva-Filho, Stevani & Desjardin	?/+	SA	[5]
***Mycenoid* lineage—Mycenaceae**			
***Mycena* species**			
Sect. *Aspratiles*			
1. *M. aspratilis* Maas Geest. & de Meijer	?/+	SA, CA	[48]
2. *M. lamprocephala* C. B. Soares & J.S. Oliveira	+/+	SA	[49]
3. *M*. *lacrimans* Singer	?/+	SA	[50]
Sect. *Basipedes*			
4. *M. illuminans* Henn. = *M. bambusa* Kawam. *nom. inval*.	?/+	MS, JP	[51,52,53,54,55]
5. *M. nocticaelum* A.L.C. Chew & Desjardin	+/+	MS	[20]
6. *M. stylobates* (Pers.: Fr.) P. Kumm. = *M. dilitata* (Fr.: Fr.) Gillet	+/+	EU, NA, CA, JP, AF	[56] [57]
Sect. *Calodontes*			
7. *M. cahaya* A.L.C. Chew & Desjardin	+/+	MS	[58]
8. *M. luceata* A. Cortés-Pérez, Guzm.-Dáv. & Ram.-Cruz	?/+	CA	[59]
9. *M. luciferina* A. Cortés-Pérez, Guzm.-Dáv. & Ram.-Cruz	?/+	CA	[59]
10. *M. lucinieblae* A. Cortés-Pérez, Ram.-Cruz & Guzm.-Dáv.	+/−	CA	[59]
11. *M. luxmanantlanensis* A. Cortés-Pérez, Ram.-Cruz & Guzm.-Dáv.	+/+	CA	[59]
12. *M*. *pura* (Pers.:Fr.) P. Kumm.	+/+ (lamellae)	EU, NA, SA, JP	[56,60]
13. *M*. *rosea* (Bull.) Gramberg	+/−	EU	[60]
14. *M. seminau* A.L.C. Chew & Desjardin	+/+	MS	[58]
15. *M. sinar* A.L.C. Chew & Desjardin	+/+	MS	[58]
16. *M. sinar* var. *tangkaisinar* A.L.C. Chew & Desjardin	+/+	MS	[58]
17. *M. sophiae* A. Cortés-Pérez	+/−	CA	[59]
18. *M. stevanii nom. prov.*	+/+	SA	Desjardin et al. (pers. obs.)
Sect. *Citricolores*			
19. *M. citricolor* (Berk. & M.A. Curtis) Sacc. = *Omphalia flavida* Maubl. & Rangel	+/−	SA, CA	[23,61]
Sect. Crocatae			
20. *M. crocata* (Schrad.) P. Kumm.	+/+	EU, JP	[62]
Sect. *Exornatae*			
21. *M. chlorophos* (Berk. & M.A. Curtis) Sacc. = *M. cyanophos* (Berk. & M.A. Curtis) Sacc.	+/+	MS, JP, PA	[20,53]
22. *M. deeptha* Aravind. & Manim.	+/−	MS	[63]
23. *M. discobasis* Métrod	?/+	SA, AF	[46]
24. *M. margarita* (Murr.) Murr.	+/+−	NA, CA, SA	[48] de Meijer (pers. comm.) N. Menolli Jr. (pers. comm.)
Sect. *Euspeirea*			
25. *M. guzmanii* A. Cortés-Pérez, Desjardin & B.A. Perry	+/+	CA	[64]
Sect. *Fragilipedes*			
26. *M*. *deusta* Maas G. & de Meijer	?/+	SA	de Meijer (pers. comm.)
27. *M. jingyinga* C.-C. Chang, C.-Y. Chen, W.-W. Lin & H.-W. Kao	+/−	CH	[65]
28. *M. luguensis* C.-C. Chang, C.-Y. Chen, W.-W. Lin & H.-W. Kao	+/−	CH	[65]
29. *M. polygramma* (Bull.: Fr.) S.F. Gray = *M. parabolica* (Fr.) Quél. *sensu* Ricken	+/−	EU, NA, JP, AF	[23,56,60]
30. *M. propria* Maas G. & de Meijer	+/+	SA	Desjardin et al. (pers. obs.)
31. *M. silvaelucens* B.A. Perry & Desjardin	?/−	MS	[48]
32. *M. stellaris* Har. Takah., Taneyama & A. Hadano	+/+	JP	[14]
33. *M. venus* C.-C. Chang, C.-Y. Chen, W.-W. Lin & H.-W. Kao	+/−	CH	[65]
34. *M. zephirus* (Fr.: Fr.) P. Kumm.	+/+ (lamellae)	EU	[56,60]
Sect. *Galactopoda*			
35. *M. haematopus* (Pers.: Fr.) P. Kumm.	+/+	EU, NA, JP	[26,60]
Sect. *Hygrocyboideae*			
36. *M. epipterygia* (Scop.: Fr.) S.F. Gray	+/+ (lamellae)	EU, NA, JP	[56]
Sect. *Lactipedes*			
37. *M. galopus* (Pers.: Fr.) P. Kumm.	+/+ (lamellae)	EU, NA, JP	[23,56,60]
Sect. *Mycena*			
38. *M. gombakensis* A.L.C. Chew & Desjardin	+/+	MS	[20]
39. *M. inclinata* (Fr.) Quél. = *M. galericulata* var. *calopus* (Fr.) P. Karst.	+/−	EU, NA, AF	[3]
40. *M*. *maculata* P. Karst.	+/−	EU, NA, AF	[60]
41. *M. tintinnabulum* (Fr.) Quél.	+/−	EU	[66]
Sect. *Nigrescentes*			
42. *M. luxfoliicola* A. Cortés-Pérez, Desjardin & Ram.-Cruz	+/+	CA	[64]
Sect. *Nodosae*			
43. *M. deformis* Maas G. & de Meijer	+/−	SA	[67]
Sect. *Roridae* (= *Roridomyces* Rexer)			
44. *M*. *aff*. *albororida* Maas G. & de Meijer	?/+	SA	de Meijer (pers. comm.)
45. *M. irritans* E. Horak	−/+	AU	[68]
46. *M. lamprospora* (Corner) E. Horak = *M. rorida* var. *lamprospora* Corner	−/+ (spores)	MS, AU	[54,69]
47. *M. pruinoso-viscida* Corner	+/+	MS	[20,53,54]
48. *M. pruinoso-viscida* var. *rabaulensis* Corner	?/+ (spores)	AU	[53,54]
49. *M. rorida* (Fr.) Quél.	+/−	EU, NA, SA, JP	[70]
50. *M. sublucens* Corner	−/+	MS	[53]
51. *Roridomyces phyllostachydis* Karun., Mortimer & Axford	+/+	MS	[71]
52. *R. viridiluminus* Karun., Dauner & Mortimer	+/+	CH	[72]
Sect. *Rubromarginatae*			
53. *M. coralliformis* A.L.C. Chew & Desjardin	+/−	MS	[20]
54. *M. cristinae* J.S. Oliveira	+/+	SA	[73]
55. *M. fulgoris* A. Cortés-Pérez & Desjardin	?/+	CA	[64]
56. *M. lumina* A. Cortés-Pérez, Desjardin & A. Rockefeller	+/+	CA	[64]
57. *M. luxcoeli* Corner	?/+	JP	[53]
58. *M. noctilucens* Kawam. ex Corner	+/+	MS, PA	[20,53,54]
59. *M. noctilucens* var. *magnispora* Corner	?/+	PA	[54]
60. *M. olivaceomarginata* (Massee apud Cooke) Massee = *M. avenacea* (Fr.) Quél.	+/−	EU, NA	[3]
61. *M. singeri* Lodge	?/+−	SA, CA	[46]
Sect. *Sacchariferae*			
62. *M. asterina* Desjardin, Capelari & Stevani	+/+	SA	[46]
63. *M. discogena* Singer	?/+	PA	Perry (pers. obs.)
64. *M. kentingensis* Shih, Chen, Lin & Kao	+/+	CH	[74]
65. *M. lazulina* Har. Takah., Taneyama, Terashima & Oba	+/+	JP (possibly AF)	[14]
66. *M. perlae* A. Cortés-Pérez, Desjardin & A. Rockefeller	?/+	CA	[64]
Sect. *Sanguinolentae*			
67. *M. nebula* A. Cortés-Pérez, Desjardin & A. Rockefeller	?/+	CA	[64]
68. *M. sanguinolenta* (Alb. & Schwein.) P. Kumm.	+/+ (lamellae)	EU, NA, JP	[56]
Sect. *Supinae*			
69. *M. fera* Maas Geest. & de Meijer	+/+	SA	[46] de Meijer (pers. comm.)
70. *M. luxarboricola* Desjardin, B.A. Perry & Stevani	?/+	SA, CA	[48,75]
71. *M. globulispora* Maas Geest. & de Meijer	+/+	SA	[64,67]
72. *M. oculisnymphae* Desjardin, B.A. Perry & Stevani = *M. aff. abieticola* Singer, reported in [48]	?/+	SA	[67]
Incertae Sedis			
73. *Mycena daisyogunensis* Kobayasi	?/+	JP	[76]
74. *M. luxaeterna* Desjardin, B.A. Perry & Stevani	+/+	SA	[48]
75. *M. luxperpetua* Desjardin, B.A. Perry & Lodge	+/+	CA	[48]
76. *M. pseudostylobates* Kobayasi	+/?	JP	[76]
77. *M. roseoflava* G. Stev.	?/+	AU	[77]
Manipularis group			
78. *Filoboletus pallescens* (Boedijn) Maas. Geest. = *Poromycena pallescens* Boedijn	?/+	MS	[78]
79. *Filoboletus yunnanensis* P.G. Liu	?/+	CH	[79]
80. *Mycena manipularis* (Berk.) Métrod *nom. inval*. [non *M. manipularis* (Berk.) Sacc.] = *Poromycena manipularis* (Berk.) Heim = *Filoboletus manipularis* (Berk.) Singer = *Polyporus mycenoides* Pat.	+/+	MS, PA, CH, AU	[20,53,80,81]
81. *Mycena manipularis* var. *microporus* Kawam. ex Corner *nom. inval*. = *Polyporus microporus* Kawam. *nom. inval*.	?/+	PA	[53]
82. *Poromycena hanedai* Kobayasi = *Polyporus hanedai* Kawam. *sensu* Kobayasi *nom*. *inval*. (not *Polyporus hanedai* Kawam. 1954) = *Mycena flammifera* Har. Takah. & Taneyama (probably a superfluous epithet, representing *Poromycena hanedai*)	+/+	JP	[76] (see [52]) [14]
*Favolaschia* species			
83. *Favolaschia pezizaeformis* (Berk. & M.A. Curtis) Kuntze	?/+	AU, JP, PA	[82,83]
84. *Favolaschia tonkinensis* (Pat.) Singer	?/+	CH, MS	Desjardin (pers. obs.)
85. *Favolaschia xtbgensis* Karunarathna & Nimalrathna	+/+	CH	[84]
*Panellus*/*Dictyopanus* species			
86. *Dictyopanus foliicola* Kobayasi	+/+	JP	[76,85]
87. *Dictyopanus pusillus* var. *sublamellatus* Corner	?/+	SA	[53]
88. *Panellus gloeocystidiatus* (Corner) Corner = *Dictyopanus gloeocystidiatus* Corner	?/+	JP, MS	[53,85,86]
89. *Panellus luminescens* (Corner) Corner = *Dictyopanus luminescens* Corner	?/+	MS	[20,69,86]
90. *Panellus luxfilamentus* A.L.C. Chew & Desjardin	+/−	MS, AU, AF	[20,86] [all Old World material reported as *P. pusillus*]
91. *Panellus pusillus* (Pers. ex Lév.) Burdsall & O.K. Mill. = *Dictyopanus pusillus* (Pers. ex Lév.) Singer = *Polyporus rhipidium* Berk.	+/+	NA, SA, AF, CH	[22,81,87]
92. *Panellus stipticus* (Bull.: Fr.) Karst. = *Panus stipticus* (Bull.) Fr.	+/+−	NA, SA, CA, AU, AF	[3,23,88,89] European and Japanese populations are non-luminescent.
*Resinomycena* species			
93. *Mycena luxfoliata* Har. Takah., Taneyama & Terashima (described as a *Mycena*, but probably represents a *Resinomycena*)	+/−	JP	[14]
94. *Resinomycena fulgens* Har. Takah., Taneyama & Oba	?/+	JP	[14]
95. *Resinomycena petarensis* Desjardin, B.A. Perry & Stevani	+/−	SA	[67]
*Cruentomycena* species			
96. *Cruentomycena orientalis* Har. Takah. & Taneyama	+/+	JP	[90,91]
**Excluded, Doubtful, and Insufficiently Known Taxa**			
1. *Collybia cirrhata* (Schumach.) P. Kumm.	?/+	EU, NA, JP	[3]
2. *Collybia tuberosa* (Bull.) P. Kumm.	?/+	EU, NA, JP	[3]
3. *Flammulina velutipes* (Curtis) Singer = *Collybia velutipes* (Curtis) P. Kumm. [a non-luminescent species]		EU, NA, JP	[92]
4. *Fungus igneus* Rumph. *nom. inval*.	?/+	MS	[3]
5. *Gerronema glutinipes* Pegler	?/+	AF	[81]
6. *Locellina illuminans* Henn. (not *Mycena illuminans* Henn.)	?/+	MS	[3,93]
7. *Locellina noctilucens* Henn. (not *Mycena noctilucens* Henn.)	?/+	AU	[3,94]
8. *Marasmius phosphorus* Kawam. *nom. inval*	?/+	JP	[52]
9. *Mycena bambusa* Kawam. *nom. inval.*	?/+	JP	[52]
10. *Mycena citrinella* var. *illumina* Kawam. *nom. inval*.	?/+	JP	[22]
11. *Mycena microillumina* Kawam. *nom. inval*.	?/+	JP	[52]
12. *Mycena phosphora* Kawam. *nom. inval*.	?/+	JP	[52] [22]
13. *Mycena photogena* Komin. *nom. inval*.	?/+	JP	[22]
14. *Mycena yapensis* Kawam. *nom. inval*.	?/+	JP	[54]
15. *Omphalia martensii* Henn.	?/+	MS	[3]
16. *Omphalia noctilucens* Rick	?/+	SA	[95]
17. *Panus incandescens* Berk. & Broome	?/+	AU	[3]
18. *Pleurotus emerci* Berk. *nom. inval.*	?/+	?	[3]
19. *Pleurotus lux* Hariot	?/+	PA	[3]
20. *Pleurotus prometheus* Berk. & M.A. Curtis = *Pleurotus djamor* (Rumph. ex Fr.) Boedijn [a non-luminescent species]	?/+	CH	[3]
21. *Polyporus noctilucens* Lagerh.	?/+	AF	[3]
22. *Xylaria hypoxylon* (L.) Grev.	+/−	NA	[96]
23. Xylariales undetermined genus/species	+/−	CA	[97]
All brown-spored agarics, boletes, polypores, corticioid fungi, gasteromycetes, and ascomycetes reported in Table III of Wassink [3] and parts A.2–A.3 of Wassink [4].			

Symbols “+” and “−” indicate presence or absence of bioluminescence, respectively, while “?” indicates presence/absence currently unknown. *1. Taxonomic synonyms are listed only if they were reported as luminescent in the published literature. *2 Distributions of luminescence in the species reported in the literature. This does not represent the global distribution of each species listed. If we consider a report unreliable, we have not included it. Europe (EU), North America (NA), South America (SA), Central America and the Caribbean region (CA), the Pacific islands (PA), China (CH), Japan (JP), Malesia, South Asia, and Southeastern Asia (MS), Australasia including Papua New Guinea and New Caledonia (AU), Africa (AF). *3. Citations where bioluminescence was reported. These are not necessarily the first or only reports of luminescence. *4 Predicted to have luminescent basidiomes: *Omphalotus mexicanus* Guzmán & V, Mora—CA; *Omphalotus olivascens* var. *indigo* Moreno, Esteve-Rav., Pöder & Ayala—CA; *Pleurotus olivascens* Corner—MS.

The taxonomy and phylogeny of *Armillaria* is well known [98,99,100,101] with 74 species recognized in Species Fungorum (www.speciesfungorum.org, accessed on 20 December 2024). Significant strides have been made in the past fifteen years in our understanding of the diversity of bioluminescent *Armillaria* species. Desjardin et al. [2] reported only five *Armillaria* species as luminescent. Herein, we report 14 species as bioluminescent with most of the additions provided by Mihail [38] in her research on the bioluminescence dynamics of North American *Armillaria*. Most species of *Armillaria* form basidiomes with a conspicuous partial veil. Those lacking a partial veil have been transferred to the genus *Desarmillaria*, wherein *D. tabescens* has been shown to have luminescent mycelium [40], while *D. ectypa* has luminescent mycelium, young rhizomorphs, and fruitbodies [43]. Interestingly, the gasteroid fungus *Guyanagaster* [102] is basal to *Armillaria* and *Desarmillaria* in a well-supported clade sister to the remaining members of the Physalacriaceae, all of which are non-luminescent [98]. The luminescent properties of *Guyanagaster* have not been determined, although its genome contains two genes of CAC (i.e., *hisps* and *h3h*) and a truncate *luz* gene that encodes the luciferase [13].

Until recently, only the mycelium, mycelial fans, and rhizomorphs of *Armillaria* have been reported as luminescent, a phenomenon known historically as foxfire [2]. The basidiomes have consistently been reported as non-luminescent, although light emission can be achieved by the addition (spraying) of an extract of fungal luciferin [103]. Herein, we report that two species, viz., *A. limonea* from New Zealand [41] and an undetermined species from Brazil (Figure 1B), form pilei that exhibit bright luminescence, while the lamellae of *D. ectypa* are weakly luminescent [43]. There are several additional reports of luminescent *Armillaria* species online, but the identification is unsubstantiated and voucher material is not available; they are not included in this report.

### 2.3. Mycenoid Lineage (Mycenaceae)

Since 2008, the greatest advances in our knowledge of the diversity of luminescent fungi have been made in the mycenoid fungi lineage (see Table 1). These fungi typically form small stipitate-pileate basidiomes with lamellate or poroid hymenophores (spore-bearing surfaces) and are saprotrophic white rot decomposers or, rarely, plant pathogens [2]. Some are known to produce luminescent mycelium but non-luminescent basidiomes, while most luminescent mycenoid species emit light from both their mycelium and basidiomes (Figure 1C). Reports of species with luminescent basidiomes but a non-luminescent mycelium are most likely erroneous (see Table 1). Additionally, there is a great variation in which part of the basidiomes emit light—from only the pileus, lamellae, or stipe, or various combinations of these structures.

**Figure 2:**
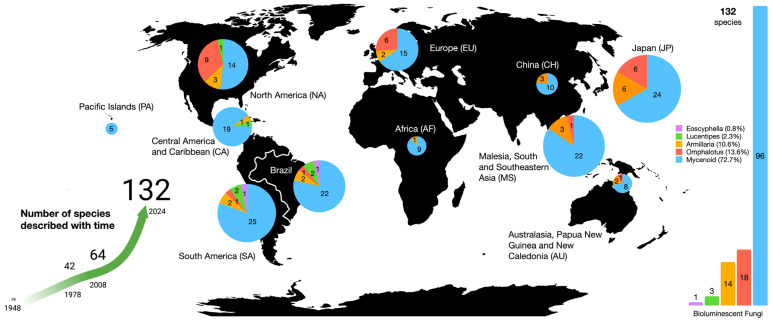
Known global distribution of bioluminescent fungi.

**Figure 3:**
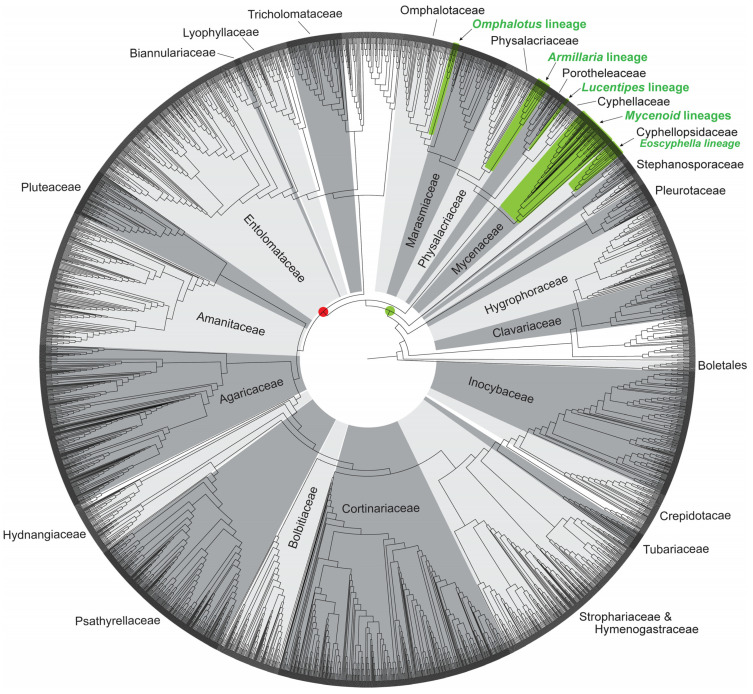
Maximum likelihood phylogeny of order Agaricales based upon *nrLSU*, *rpb2*, and *ef1-a* sequence data, with bioluminescent lineages highlighted in green. Green circle indicates origin of bioluminescence in the MRCA to the Marasmiineae clade based upon relationships of bioluminescent lineages. Red circle represents hypothesized loss of bioluminescence that rendered remaining Agaricales non-luminescent. Please see Supplementary Materials for details on data and phylogenetic reconstruction methods.

**Figure 4:**
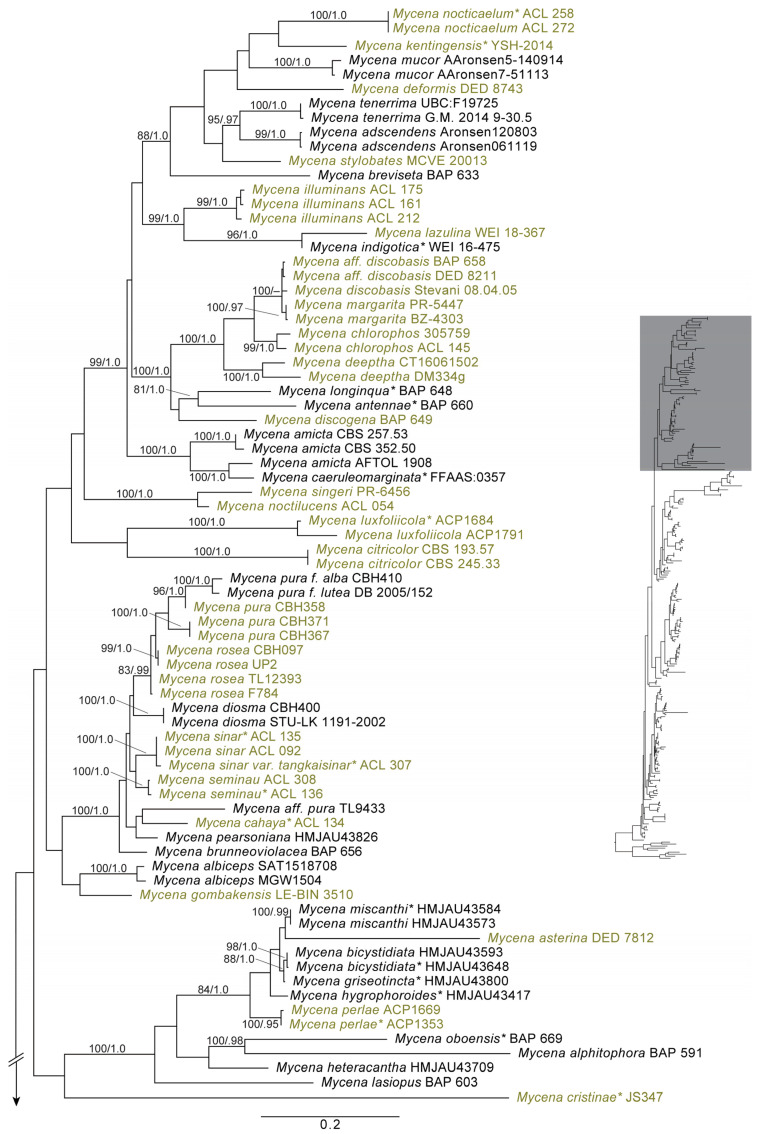

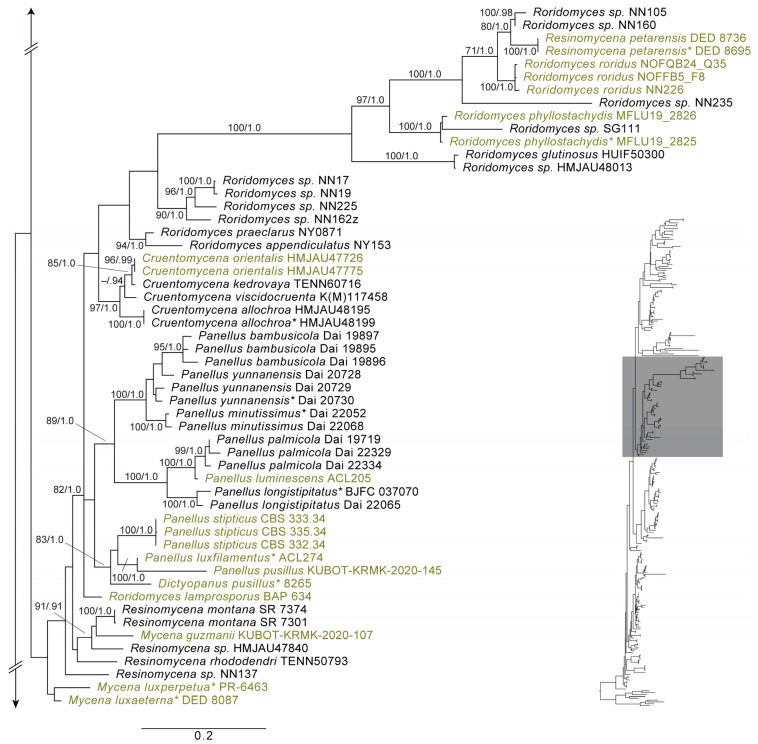

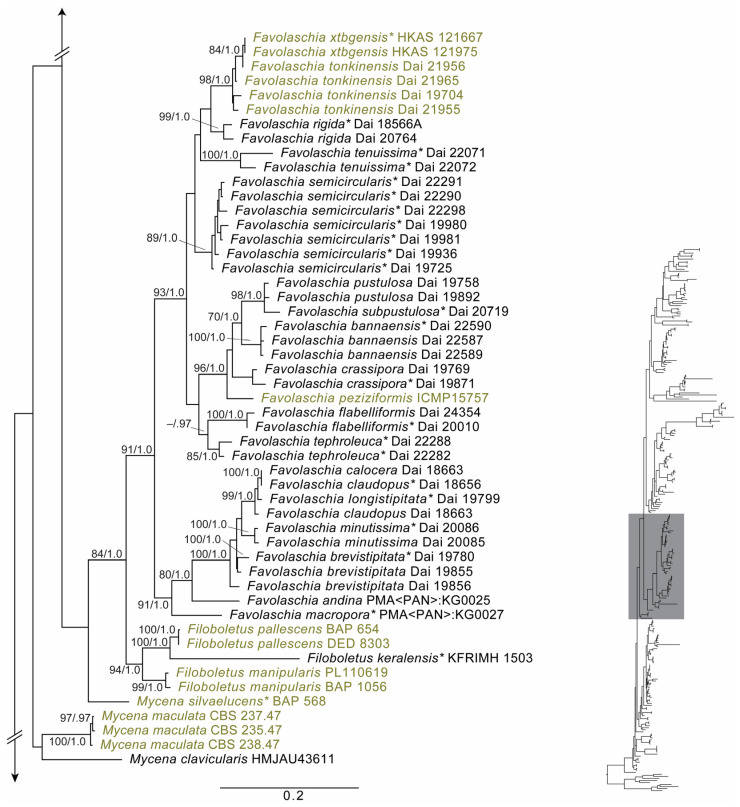

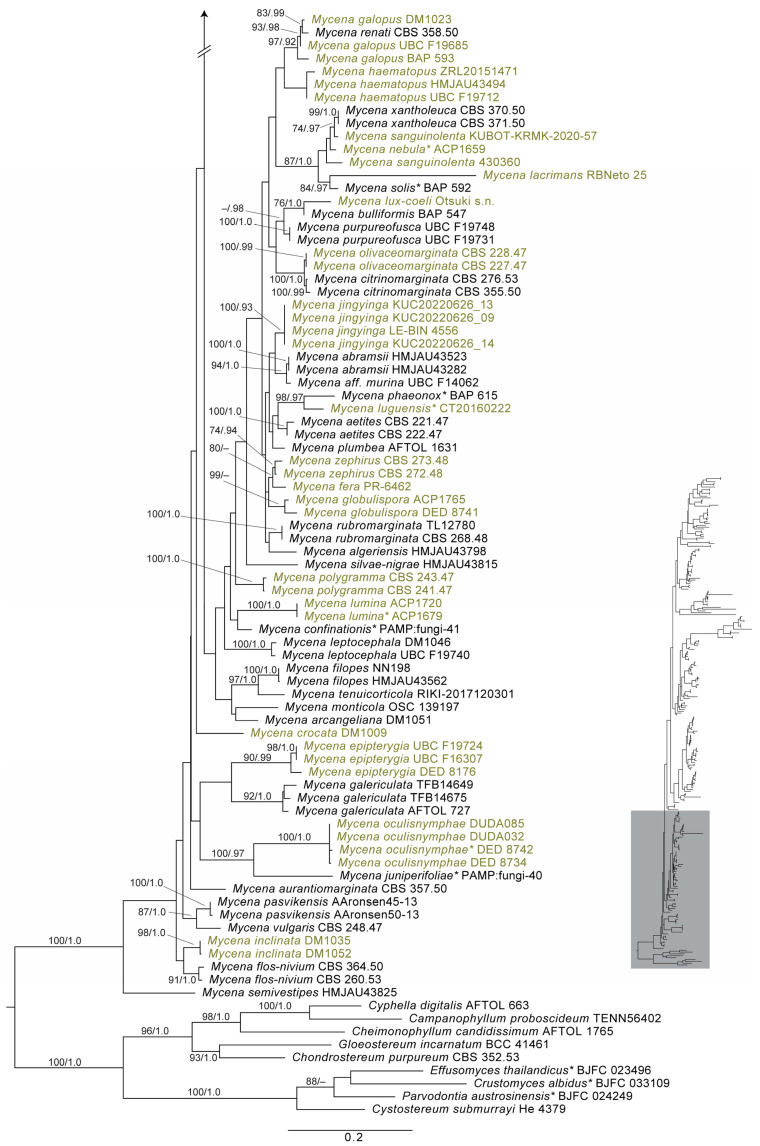
Maximum likelihood phylogeny of Mycenceae based upon *ITS* and *nrLSU* sequence data, with bioluminescent species highlighted in green font. Values separated by/refer to ML bootstrap proportions and Bayesian posterior probabilities for values over 70/0.90, respectively. Sequences derived from type specimens are designated with an asterisk *. Please see Supplementary Materials for details on data and phylogenetic reconstruction methods.

Most luminescent mycenoid species have been described in the polyphyletic genus *Mycena*. Desjardin et al. [2] reported 45 luminescent mycenoid species (excluding the Lucentipes lineage noted below) belonging to 16 historically accepted infrageneric groups of *Mycena s.l.*, with additional species described in *Panellus*, *Dictyopanus*, *Filoboletus*, and *Poromycena*. That number has doubled in the past fifteen years to 96 species reported herein (Figure 2). Bioluminescent species of *Mycena s.s.* belong to 19 historically accepted infrageneric groups plus a few *incertae sedis* (Table 1). Additionally, we report luminescent species currently placed in *Roridomyces*, *Filoboletus*, *Poromycena*, *Favolaschia*, *Dictyopanus*, *Panellus*, *Cruentomycena*, and *Resinomycena*. Based on both the phylogenetic analyses included here (Figure 3 and Figure 4), as well as our unpublished molecular data for additional markers, all of these latter genera fall into a well-supported *Mycena s.s*. clade, and their acceptance as *Mycena* would render the latter genus monophyletic. Most of the new reports of bioluminescent mycenoid taxa are based on the work of Desjardin et al. [46,67] and Oliveira et al. [73] from Brazil, Cortés-Pérez et al. [64] from Mexico, Chew et al. [20,55,58] from Malaysia, Karunarathna et al. [71] from India, Shih et al. [74] and Chang et al. [65] from Taiwan, Terashima [14] and Oba and Hosaka [44] from Japan, and Dauner et al. [72] and Nimalrathna et al. [84] from China.

### 2.4. Lucentipes Lineage (Cyphellaceae/Porotheleaceae)

Desjardin et al. [2] included *Gerronema viridilucens* and *Mycena lucentipes* (Sect. Diversae) among the 47 taxa reported in the Mycenoid lineage. Since then, based on both unpublished and published multigene analyses, it has become apparent that these two species do not belong to the Mycenaceae *sensu stricto* (Silva-Filho et al. [5]). *Mycena lucentipes*, along with *Mycena quiniaultensis* Kauffman (reported herein as bioluminescent), is resolved as being closely related to *Mycopan scabripes* (Murrill) Redhead, Moncalvo & Vilgalys and species of *Atheniella* Redhead, Moncalvo, Vilgalys, Desjardin & B.A. Perry. In the recent analyses of Vizzini et al. [104], *Mycopan*, *M. quiniaultensis*, and *Atheniella*, along with additional segregate mycenoid genera such as *Phloeomana* Redhead and *Mycenella* (J.E. Lange) Singer, are resolved within family Cyphellaceae based upon *nrLSU* sequence data. However, in the Agaricales phylogenetic reconstruction included here (Figure 3), which is based upon re-analysis of the megaphylogeny of Varga et al. [105], *Mycopan scabripes*, *Gerronema viridilucens*, and several *Atheniella* taxa are resolved in a clade that is basal to a sampling of taxa representing the Porotheleaceae. Additional markers and taxon sampling will likely be required to further resolve the family placement of these taxa. Our unpublished analyses, as well as those of Silva-Filho et al. [5] and Vizzini et al. [104], all suggest that both *M. lucentipes* and *M. quiniaultensis* should likely be treated in the genus *Mycopan* Redhead, Moncalvo & Vilgalys. *Gerronema viridilucens*, while closely related to *Mycopan* in our unpublished analyses, is consistently resolved as a distinct lineage and should be recognized as a new genus. Both *M. lucentipes* and *G. viridilucens* form luminescent mycelium and basidiomes, and show similar basidiome macro- and micromorphology. These taxa differ in that *G. viridilucens* has inamyloid basidiospores, a few of which show golden resinous contents, only the lamellae are luminescent, and it grows on living *Eugenia* trees [45], while *M. lucentipes* (Figure 1D) forms distinctly amyloid basidiospores lacking pigmented contents, only the stipes emit light, and it grows on a variety of rotting dicotyledonous sticks and roots [46]. *Mycena quiniaultensis* and *Mycopan scabripes* share micromorphological similarities with *M. lucentipes*. Rockefeller [47] has reported the basidiomes of *M. quiniaultensis* as being luminescent, whereas there are no reports on the luminescence of *Mycopan scabripes*. The mycelium of *G. viridilucens* was used to study the single origin of luminescence in fungi [7], and for the development of a toxicological bioassay to evaluate the toxicity of inorganic and organic compounds to basidiomycetes [8,106,107,108,109,110]. Luminescent basidiomes of *M. lucentipes* graced a 2018 set of US postage stamps displaying bioluminescent life [111], representing the first time a mushroom was the exclusive feature of a US stamp. 

### 2.5. Eoscyphella Lineage (Cyphellopsidaceae)

Continuing field exploration in southern Brazil has uncovered an interesting cyphelloid species that represents a new genus and new species belonging to the Cyphellopsidaceae (syn. Niaceae) [5]. It forms tiny (0.3–0.5 mm long), pendant, vasiform to urceolate basidiomes on the bark of living *Solanum schwartzianum*, with light emitted from the margin of the receptacle. This represents the first known light-emitting cyphelloid species and a previously unknown bioluminescent lineage. The recent discovery of *Eoscyphella* and a fifth bioluminescent lineage within the Agaricales underscores the need for continued exploratory work into documenting fungal bioluminescence.

### 2.6. Excluded, Doubtful, Insufficiently Known, and Misdiagnosed Taxa

There remain several poorly known species once reported as bioluminescent, primarily invalid species reported from Japan by Haneda [52], epithets compiled by Wassink [3], or misinterpretations of weak chemiluminescence [92,96]. We add to this list several misdiagnosed taxa or erroneous reports.

Terashima et al. [14] described two new species of *Marasmiellus* that they reported as luminescent, *M. lucidus* and *M. venosus*. However, the two ITS sequences submitted to Genbank (#’s OP459424 and OP459425, respectively) are 99.3% similar to each other and show the closest relationship to *Omphalotus japonicus* (99% identical, 100% coverage). We recognize these as misdiagnosed taxa belonging to the *Omphalotus* lineage, and not closely related to *Marasmiellus*.

The first reports of luminescence in an Ascomycete were from several species of *Xylaria*. Ludwig [112] reported that the mycelium of *Xylaria hypoxylon* (L.) Grev. in rotten wood was luminous, while Crié [113] noted a similar phenomenon for the mycelium of *X. polymorpha* (Pers.) Grev. Several decades later, Molisch [114] tested these observations with *X. hypoxylon* and *X. cookei* Lloyd and was unable to confirm luminescence in pure mycelial cultures. As noted by Buller [115], Molisch grew cultures of *X. hypoxylon* for four years, and during that period he was unable to note any light emission from either the mycelium or fruit bodies. In contradiction to this, Guéguen [116] was able to confirm Ludwig’s observations from the mycelium of *X. hypoxylon* grown on several media. It should be noted, however, that Guéguen stated the luminescence “seemed very feeble and in no way comparable in intensity with that one observes so frequently during the warm season on fish and other marine animals exposed to the air. The glow of *Xylaria* is white tinged with blue, and one can only perceive it clearly in complete darkness”. The latter observation suggests that the light emitted was not true fungal bioluminescence, which is yellowish green. Guéguen’s observations and the more recent report of bioluminescence in *X. hypoxylon* (Ascomycota, Xylariales) from the United Kingdom [117] most likely represent either ultraweak chemiluminescence or simple light refraction of a white surface at night. Bioluminescence is the emission of light by living organisms, driven by an enzymatic reaction that is controlled and regulated by the organism. In fungi, this process is linked to the Caffeic Acid Cycle and involves luciferase enzymes (see below). In contrast, ultraweak chemiluminescence is not controlled by the organism but rather originates from a non-enzymatic process, often involving reactive oxygen species (ROS) or lipid peroxidation. This type of emission is weak, short-lived, and usually not visible to the naked eye. It occurs randomly as a byproduct of oxidative stress or metabolic reactions and does not play a biological role. 

In addition, a report of an undetermined luminescent member of the Xylariales from Costa Rica [97] is based on the observation of a luminescent mycelium in palm roots on which xylariaceous fruitbodies occurred, but with no indication that these fruitbodies were luminescent and no photographs were published. The authors did not provide data proving that the luminescent mycelium observed belonged to the species that formed the xylariaceous fruitbodies. To our knowledge, there are no well-documented and substantiated reports of bioluminescence in the Ascomycota, nor reports that ascomycetes contain the gene clusters required for fungal bioluminescence. The reference genome of *X. hypoxolon* can be found in the NCBI database (GCA_902806585.1). However, when searching for the luciferase sequence, for instance, of *Neonothopanus gardneri* in the genome of this ascomycete fungus, no homologous genes can be found.

## 3. Distribution of Bioluminescent Fungi

Bioluminescent fungi have been reported from all continents except Antarctica (Figure 2). Until recently, documenting the occurrence of these fungi was often a consequence of serendipitous nocturnal encounters as opposed to focused diversity studies. Our knowledge of their distribution is mostly a direct reflection of the peregrinations of mycologists, and is undoubtedly incomplete. Only in the past fifteen years (see Table 1) have researchers specifically searched for bioluminescent species through nighttime collecting or by testing day-collected specimens with luminometers or photographing under entirely dark conditions with digital cameras capable of long exposure times (e.g., 8 min or longer) and high sensitivity settings. 

The geographical regions from which populations of luminescent species have been reported is provided in Table 1, but does not reflect the global distribution of each species included. Some listed species occur in regions not reported here, but their luminescent properties have not been recorded from such areas. For example, many of the temperate species of *Mycena* listed occur in China, but we have no information that these taxa have been observed to be luminescent there. With that caveat, in order of highest diversity, 36 species of bioluminescent fungi have been reported from Japan, 31 from South America (mostly from Brazil), 27 from North America, 26 from Malesia, South Asia, and Southeast Asia, 23 from Europe, 21 from Central America, 13 from China, 11 from Australasia, Papua New Guinea, and New Caledonia, 10 from Africa, and 5 from the Pacific Islands (Figure 2). 

It is difficult to assess which type of habitat or substrate hosts most luminescent species as they are found in temperate, subtropical, and tropical habitats and on a multitude of substrates. In our experience, the greatest diversity of luminescent euagarics occurs in woody or leafy substrates in subtropical closed canopy forests with high plant diversity. As indicated above, the highest species richness is among the mycenoid species, which grow in both temperate and tropical habitats, with 24 species recorded from Japan, 22 from Malesia and southern Asia, 22 from Brazil, 19 from Central America, 15 from Europe, and 14 from North America. The paucity of luminous species reported from Africa and other mycologically understudied regions is likely due to the limited amount of research on mushroom diversity (especially at night) from these regions and not a true reflection of their occurrence. Likewise, a concerted effort to document the luminescent fungi of China and Taiwan will substantially increase the totals reported here. The increasing use of environmental DNA (eDNA) in metabarcoding studies of fungal diversity, especially from woody and leafy substrates, also promises to greatly increase our understanding of the distribution of known bioluminescent taxa. Future studies taking a metabarcoding approach also have the potential to provide insight into additional preferred substrates of luminescent taxa, and may shed light on the potential ecological roles of fungal bioluminescence. 

*Omphalotus* lineage representatives are relatively evenly distributed across the globe with 2–4 species reported from each region. Most are region-specific, although *Neonothopanus nambi* shows an amphi-Pacific distribution. *Armillaria* is a mainly north-temperate genus, with the highest diversity of luminescent species in North America (9), Japan (6), and Europe (6). Finally, the Lucentipes lineage is known only from Brazil, Puerto Rico, and western North America, while the *Eoscyphella* lineage is known only from southern Brazil. As more focused fieldwork and lab work are conducted, we suspect many additional new bioluminescent species will be discovered and many species currently considered non-luminescent will be verified as luminescent, as demonstrated in the recent study by Heinzlemann et al. [62] documenting both observable luminescence and the necessary genetic architecture in *Mycena crocata*.

## 4. Evolution of Bioluminescence in Fungi

Results of our phylogenetic analyses (Figure 3), as well as those of previous investigators [9,13,118], indicate that bioluminescence arose a single time early in the evolution of the Agaricales, and was subsequently lost or inactivated in many taxa throughout the evolutionary history of this group. Oliveira et al. [9] provided evidence of a common substrate and enzymes in the four evolutionary lineages of bioluminescent fungi known at that time. Using hot (substrate) and cold (enzyme) extraction methods, these authors performed cross-reactions between exemplar bioluminescent species from four lineages and demonstrated that all combinations resulted in measurable light emission (bioluminescence), supporting the hypothesis of an identical enzymatic mechanism operating in all bioluminescent fungal lineages known at that time. Cross-reactions of representatives from all four lineages with extracts from non-luminescent control species failed to produce measurable light, confirming that this non-luminescent species contains neither luciferin nor the enzymes required for bioluminescence.

**Figure 5:**
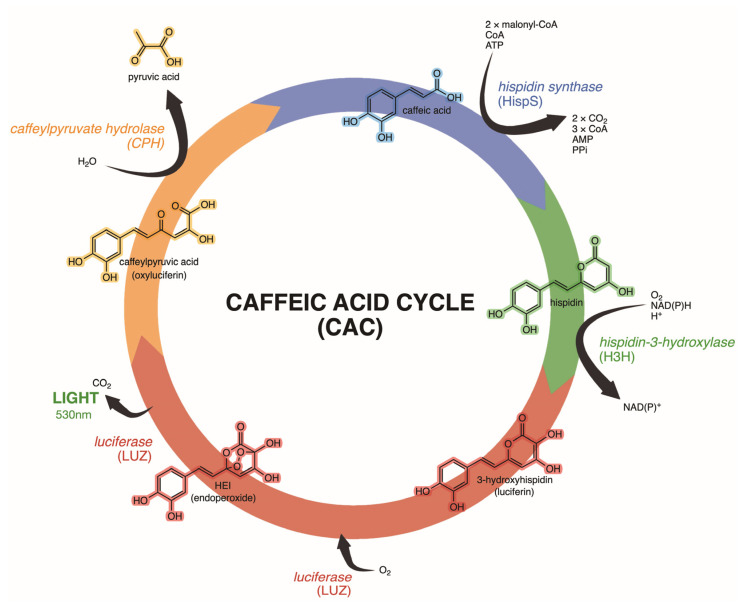
The Caffeic Acid Cycle (CAC), the biochemical pathway responsible for fungal bioluminescence.

Kotlobay et al. [13] provided identification of the fungal luciferase and three additional key enzymes that together form a biosynthetic cycle, the Caffeic Acid Cycle (CAC, Figure 5 below), to produce the fungal luciferin, 3-hydroxyhispidin, via the oxidation of the widespread plant and fungal metabolite caffeic acid. Utilizing genome and transcriptome sequence data, these authors demonstrate that the luciferase gene (*luz*), as well as the other genes involved in the pathway (*hisps* and *h3h*), are part of a conserved gene cluster in bioluminescent fungi which likely evolved a single time. These authors provide additional evidence to suggest that the primary luciferase gene arose early in the evolution of Agaricales via gene duplication. The luciferase gene (as isolated in *Neonothopanus nambi* and presumably the same in all bioluminescent fungi) has no described homologs or significant sequence similarity to conserved protein domains, and, therefore, likely represents the origin of a novel protein family. Additional duplication events are proposed to account for the origin of the *h3h* and *hisps* genes several million years later. In several bioluminescent species sampled in this work, the conserved gene cluster includes one or two additional genes, one belonging to the cytochrome P450 family, and the other (*cph*) belonging to the family of fumarylacetoacetate hydrolases. The latter gene (*cph*) is believed to encode a caffeylpyruvate hydrolase that is involved in the recycling of oxyluciferin back into caffeic acid, completing the bioluminescent pathway cycle. The results of Kotlobay et al. [13] indicate that the *cph* gene is lacking in the mycenoid lineage but may have been inserted independently into the bioluminescent gene cluster twice, once in the *Omphalotus* lineage and once in the *Armillaria* lineage (Figure 3). It has not yet been determined if *cph* or cytochrome P450 occur in the gene cluster of the *Eoscyphella* or Lucentipes lineages.

The phylogenomic analyses of Ke et al. [118], based on a concatenated supermatrix of 360 single copy orthogroups, also suggest a single origin of bioluminescence within the Agaricales dated to approximately 160 mya. The results of these authors place five bioluminescent *Mycena* species included in the analyses as sister to a Marasmioid clade (Marasmiineae) that contains species currently treated in Physalacriaceae, Omphalotaceae, Fistulinaceae, and Marasmiaceae. These analyses did not include bioluminescent taxa from the Lucentipes lineage (i.e., *Mycena lucentipes* and *Gerronema viridilucens*), and pre-date the discovery and publication of *Eoscyphella* in the Cyphellopsidaceae [5]. Ke et al. [118] suggest that the orthogroup containing luciferase (as well as *h3h* and *cph*) was present in the common ancestor to the Mycenoid and Marasmioid lineages, as well as non-bioluminescent taxa *Schizophyllum commune* and *Auricularia ampla*, predating their inclusion into the luciferase cluster. These authors state that this finding contrasts with the results of Kotlobay et al. [13], suggesting that the results of these authors indicate the luciferase gene evolved in the ancestor to the Agaricales. This interpretation is incorrect as Kotlobay et al. [13] (in their Figure 2) suggest that the luciferase gene evolved in the ancestor to the Agaricales and *Schizophyllum commune*, which is sister to *Fistulina hepatica* in their phylogenetic analyses. Ke at al. [118] also propose that the ancestral bioluminescence gene cluster consisted of *luz*, *h3h*, cyp450, and *hisps*, with *cph* located on the same chromosome (this combination was found in 14 of the 15 bioluminescent species in their study). They also suggest that the *cph* gene had been independently translocated to a position adjacent to the bioluminescent gene cluster in *Mycena kentingensis* and the ancestor to the Marasmioid clade, and has been maintained in this location by natural selection.

In addition to the single early origin of bioluminescence within fungi, there have also been multiple evolutionary losses of one or more key components of the pathway that have rendered members of the five luminescent lineages, as well as the majority of Agaricales, non-luminescent. The results of Kotlobay et al. [13] (in their Figures 1 and 2) indicate at least six independent, complete to partial gene losses from the cluster, leading to the secondary loss of bioluminescence in the *Armillaria*, *Omphalotus*, Mycenoid, and Lucentipes lineages. Within the Mycenoid lineage, which contains the majority (~73%) of described bioluminescent species, the distribution of bioluminescence is especially patchy across a phylogenetic sampling of these genera (Figure 4), suggesting the loss of one of more required genes multiple times independently. A similar pattern can be seen in the *Omphalotus*, Lucentipes, and *Eoscyphella* lineages, all of which are characterized by predominantly non-bioluminescent species. Ke et al. [118] propose a scenario for gene cluster loss within the Agaricales. Due to a lack of synteny in the genes surrounding the bioluminescent gene cluster in both the Mycenoid and *Omphalotus* lineage species included in their analyses, these authors suggest that (at least within *Mycena*) the cluster is located within a “highly dynamic” genomic partition and is therefore prone to loss through gene alteration. In the *Armillaria* lineage, which displays high levels of synteny surrounding the bioluminescence genes, the cluster is suggested to be in a slowly evolving region of the genome and is therefore less prone to losses and other chromosomal alterations. For this reason, the cluster has remained highly conserved across *Armillaria*, resulting in most (or all) of these species retaining bioluminescent properties. The results of our analyses suggest that a single, partial to complete loss of genes making up the bioluminescence cluster are all that was required to render the remainder of the Agaricales outside the Marasmiineae non-bioluminescent (Figure 3).

Within the Mycenaceae, bioluminescence has been observed in 96 species currently treated in *Mycena*, *Cruentomycena*, *Dictyopanus*, *Favolaschia*, *Filoboletus*, *Panellus*, *Poromycena*, *Resinomycena*, and *Roridomyces*. Our phylogenetic analyses, based upon *nrLSU* and *ITS* sequence data (Figure 4), as well as unpublished analyses including additional markers and previously published analyses [5], do not support existing infrageneric classifications within *Mycena* and render the genus non-monophyletic as currently circumscribed. In our analyses, all of the latter genera listed above are embedded in a well-supported *Mycena s.s*., with several genera (*Cruentomycena*, *Favolaschia*, *Filoboletus*) forming well-supported groups within *Mycena s.s*. as sampled here. The remaining genera represented by more than a single representative sequence fall out in weakly supported clades within *Mycena s.s*. (*Roridomyces*, *Panellus*), or are resolved in non-monophyletic clades (*Resinomycena*). The type of the genus, *Mycena galericulata* (Scop.) Gray, is resolved towards the base of the Mycenaceae lineage. The logical solution to this situation is the recognition of all these species within *Mycena sensu lato*, rendering the genus monophyletic (this will be addressed in a forthcoming manuscript). Although our taxonomic sampling is incomplete, the phylogenetic analyses included here (Figure 4) suggest that there have been multiple independent evolutionary losses of one or more key components of the bioluminescent pathway within the Mycenaceae, or that many species within this lineage have not yet been observed to produce bioluminescent basidiomes and/or hyphae. As stated above, we suspect that many species currently considered non-luminescent will be verified as luminescent with continued field documentation and research.

## 5. Metabolic Pathway of Fungal Bioluminescence

The understanding of fungal bioluminescence mechanisms has advanced through insights from studying bioluminescent beetles and bacteria. In the late 1880s, Raphaël Dubois conducted experiments with the bioluminescent organs of the West Indies beetle, coining the terms luciferin and luciferase [119]. About eighty years later, Airth and McElroy [120] proposed that fungal bioluminescence required luciferin, a NAD(P)H-dependent reductase, molecular oxygen, and luciferase. By the end of the 1980s, the existence of a fungal luciferase remained controversial due to the complexity of the system. Further investigations by Airth and Foerster [121] led to the discovery of light emission by mixing cold (source of proteins) and hot (source of luciferin) extracts from different fungi. These researchers identified two essential components for the light reaction: a soluble protein and a membrane protein. They proposed a two-step mechanism involving an unknown electron acceptor reduced by NAD(P)H, which then reacted with a membrane-bound luciferase and molecular oxygen to emit light. This mechanism, similar to bacterial bioluminescence, suggested a shared process among fungi, although fungal bioluminescence did not require a reduced flavin mononucleotide, a flavin adenine dinucleotide, or an aliphatic long-chain aldehyde. Despite these findings, it took nearly sixty years to recognize the cyclic nature of fungal bioluminescence [119].

The discovery of new bioluminescent fungal species and successful replication of Airth and Foerster’s [121] results suggested a common bioluminescence mechanism across fungi. Cross-reactions between extracts of various fungi showed light emission, indicating fungal luciferin and luciferase were common in bioluminescent fungi metabolism [7,9]. Subsequent experiments using Oliveira’s protocol identified hispidin as a precursor to fungal luciferin, and found higher quantities of hispidin in non-luminous fungi such as *Pholiota squarossa* [122]. The role of hispidin was confirmed through bioluminescent assays, leading to the identification of fungal luciferin as 3-hydroxyhispidin. This discovery corrected earlier assumptions, recognizing the NAD(P)H-dependent enzyme as a monooxygenase, and marked a significant advancement in understanding fungal bioluminescence, distinct from other known luciferins [119].

To understand the chemical mechanism of fungal bioluminescence, researchers identified oxyluciferin as the product of the luciferin–luciferase reaction by monitoring the reaction of synthetic luciferin with luciferases from *N. nambi* and *N. gardneri* in the presence of isotopic labelled molecular oxygen and HPLC-MS [12]. The chromatogram produced in that study showed peaks indicating unstable oxyluciferin and its degradation products, including caffeic acid, suggesting a hydrolysis step. Further experiments confirmed the biochemical mechanism involving 3-hydroxyhispidin oxidation and light emission, with subsequent studies clarifying the role of caffeic acid in hispidin biosynthesis [13].

With strong evidence for the mechanism of fungal bioluminescence, and using a cDNA library from *N. nambi*, fungal luciferase and the genes that encode for other enzymes related to fungal bioluminescence were discovered [13]. Comparative genomic and transcriptomic analyses across bioluminescent fungi revealed orthologous genes involved in the luminescence pathway [119]. A conserved gene cluster, including hispidin synthase (*hisps*), hispidin-3-hydroxylase (*h3h*), luciferase (*luz*), and caffeylpyruvate hydrolas (*cph*), was identified, supporting the hypothesis of a common ancestor for fungal bioluminescence [13]. This discovery, termed the Caffeic Acid Cycle (CAC), confirmed a common ancestor for fungal bioluminescence and established the first known eukaryotic luciferin biosynthetic pathway.

The CAC begins with caffeic acid (Figure 5), produced via the Shikimate pathway, which is converted into hispidin by *hisps*, an enzyme from the polyketide synthase (PKS) family. Hispidin is then hydroxylated by *h3h* to yield fungal luciferin (3-hydroxyhispidin). This luciferin reacts with luciferase in the presence of molecular oxygen, producing oxyluciferin (caffeylpyruvate) and a photon of light at 530 nm. Finally, *cph* acts on oxyluciferin to complete the cycle [13,119].

## 6. Functional Significance of Fungal Bioluminescence

Bioluminescence is a chemical process that requires molecular oxygen, and all luciferins are reducing agents. Therefore, it is reasonable to infer that luciferins can act as antioxidants, protecting the organism from the harmful effects of reactive oxygen species (ROS) produced during respiration and other oxygen-dependent biological processes [2,123,124,125]. Additionally, it is important to note that all known bioluminescent fungi are basidiomycetes. Therefore, it has been suggested that fungal bioluminescence could provide additional protection against the oxidative stress involved during lignin degradation [26,126,127]. This would then be the primary function of bioluminescence, although in several bioluminescent organisms, this primary function has evolved into one or more functions with ecological significance.

To fully understand fungal bioluminescence, it is essential to evaluate not only its biochemical but also its ecological role. A fact that corroborates the possible ecological nature of fungal bioluminescence is its circadian rhythm [10,128], as control implies function. Although light is emitted continuously, a circadian rhythm is observed, with the maximum output at night, peaking around 9:00 PM. Behavioral observations can provide this information, as seen with fireflies, millipedes, and dinoflagellates [129,130,131,132]. However, the selective advantages of bioluminescence in fungi still deserve further examination. In the first attempt to understand the ecological significance of fungal bioluminescence, Sivinski [133] conducted an experiment using sealed test tubes and glass jars containing forest litter and foliage covered with luminescent mycelium and *Dictyopanus pusillus* mushrooms. In his experiment, carried out in Alachua County, Florida, USA, bioluminescent and control traps were set at night and collected the next morning in areas with the presence of the bioluminescent fungus. The arthropods glued to the adhesive surfaces were removed and catalogued. The study found that traps baited with luminescent glowing fungal structures captured more arthropods than non-luminescent traps. Based on these findings, Sivinski suggested several potential ecological roles for fungal bioluminescence, including attracting spore dispersers, carnivores of fungivores, and fertilizers, repelling negatively phototropic fungivores, and acting as an aposematic signal.

Three and a half decades after Sivinski’s research, the interaction of arthropods with bioluminescent mushrooms was revisited, this time using green LED lights and acrylic mushroom replicas [10]. The experiments conducted in the Maranhão Babassu forest (“Mata dos Cocais”, Brazil) revealed that illuminated acrylic mushrooms attracted significantly more staphylinid rove beetles (Coleoptera), Hemiptera (true bugs), Diptera (flies), and Hymenoptera (wasps and ants) than dark control traps. The authors concluded that circadian control could optimize energy use for when bioluminescence is most visible, attracting insects that can aid in spore dispersal, thereby benefiting fungi growing under the forest canopy where air flow is minimal. These conclusions, as well as those of Sivinsky [133], suggest a potential selective advantage for bioluminescence in the above-ground reproductive structures where spores are produced (i.e., lamellae and pileus), but do not suggest an explanation for luminescence in the lower portions of the stipe or the mycelium (which do not produce reproductive spores). The emission of light in non-spore-producing tissues may well be linked to other ecological roles of the phenomenon. 

Nevertheless, the hypothesis that bioluminescence in some fungi may occur as an accidental result of their metabolism, rather than for any evolutionary benefit, cannot yet be excluded. The function of bioluminescence in fungi could also vary between different evolutionary lineages and between different phases of the life cycle (mycelium versus basidiomes), and might be influenced by environmental factors such as wind and the presence of insects, which could affect spore dispersal [134].

## 7. Conclusions

- All known bioluminescent fungi are mushroom-forming species of Basidiomycota from order Agaricales. The documented number of known bioluminescent fungi has more than doubled in the past 15 years from 64 to 132 species.
- Five distinct lineages of bioluminescent Agaricales are currently recognized based on molecular phylogenetic analyses. These include: Omphalotaceae (18 species), Physalacriaceae (14), Mycenaceae (96), Lucentipes lineage—Cyphellaceae/Porotheleaceae (3), and Cyphellopsidaceae (1).
- While many regions remain poorly documented for bioluminescent fungi, the areas with the most known species are Japan (36 species), South America (30), North America (27), Malesia, South Asia, and Southeast Asia (26), Europe (23), Central America (21), China (13), Australasia, Papua New Guinea, and New Caledonia (11), Africa (10), and the Pacific Islands (5).
- Recent studies have elucidated the biochemical and genetic pathways of fungal bioluminescence and suggest the phenomenon originated a single time early in the evolution of the Agaricales. Although many plants and non-bioluminescent fungi are able to biosynthesize hispidin (the fungal luciferin precursor), only bioluminescent fungi contain *h3h*, *luz*, and *cph* genes in their genomes. To date, the Caffeic Acid Cycle (CAC) is the only fully encodable eukaryotic bioluminescent system.
- Multiple independent evolutionary losses explain the absence of luminescence in many species found within the five bioluminescent lineages and in the majority of Agaricales.
- Bioluminescence in fungi may primarily function as a defense against oxidative stress. While there is strong evidence that it can serve as an ecological strategy to attract spore-dispersing insects, its role may vary between species, life cycle phases, and environmental conditions, and it might sometimes be merely an accidental metabolic byproduct.

## Data Availability

The data presented in this study are available on request from the corresponding author.

## Supplementary Materials

The following supporting information can be downloaded at: https://www.mdpi.com/article/10.3390/jof11010019/s1, Materials and methods for phylogenetic analyses used to generate Figure 3 and Figure 4; Table S1: Sequences included in Mycenaceae phylogenetic analyses (Figure 4) and corresponding Genbank accession numbers. References [135,136,137,138] are cited in the supplementary materials.
