# Supplementary material for: Diversity, Distribution, and Evolution of Bioluminescent Fungi
Source: J Fungi (Basel). 2024 Dec 31;11(1):19. doi: 10.3390/jof11010019 (PMC11766655; doi:10.3390/jof11010019)
Supplement: Supplementary file 1 [file jof-11-00019-s001.zip › jof-3353135-supplementary.pdf]

Diversity, distribution and evolution of bioluminescent fungi

Brian A. Perry, Dennis E. Desjardin, and Cassius V. Stevani

Supplementary Materials

Materials and Methods

Figure 3. Maximum likelihood phylogeny of order Agaricales. Dataset adapted from Agaricomycotina data of Varga et al. [105] and consists of sequence data for up to three nuclear markers (*nrLSU, rpb2* and *ef1-a*) for of total of 5738 nucleotides for 3344 taxa. Sequences of the Boletales taxa included were used as the outgroup for rooting purposes. Sequences for all other non-Agaricales taxa present in original dataset were trimmed prior to analyses. Maximum likelihood analyses were run in RAxML 8.2.12 [135], as implemented in the CIPRES Science Gateway [136], under a GTRCAT model and consisted of four alternative runs using default parameters estimated independently by the program for each of the three gene partitions. As in the analyses of Varga at al. [105], the deeper nodes in the phylogeny were constrained during the analysis through the use of a backbone constraint tree based upon phylogenetic analysis of 104 Agaricomycotina genomes. The backbone constraint phylogeny of Varga et al. was adapted to reflect only those taxa included in the present analysis.

Figure 4. Maximum likelihood phylogeny of Mycenaceae. The dataset consists of sequence data for two markers (*ITS* and *nrLSU*) for a total of 2532 nucleotides for 267 voucher collections, and includes all Mycenaceae sequences available in GenBank (GB) at the time of writing for which both *ITS* and *nrLSU* data were generated from the same voucher collection. Sequences of additional bioluminescent species/vouchers represented by only *ITS* or *nrLSU* were also included

to generate the largest phylogenetic sampling of bioluminescent Mycenaceae to date. Sequences of species from Cyphellaceae and Porotheleaceae were included as the outgroup for rooting purposes. Please see Table S1 for a complete list of included sequences and corresponding GB numbers. Maximum likelihood analyses were run in RAxML 8.2.12 [135], as implemented in the CIPRES Science Gateway [136] (Miller et al., 2010), under a GTRCAT model and consisted of 500 alternative runs using default parameters estimated independently for each gene partition, with node support estimated by 500 RAxML bootstrap replicates. Bayesian analyses were performed using Metropolis Coupled MCMC methods as implemented in MrBayes 3.2.6 [137] under an GTR+I+G model of sequence evolution as determined under the Bayesian Information Criterion in PAUP*4.0a.167 [138]. Bayesian analyses consisted of two parallel searches, run for 40 million generations, initiated with random starting trees and default chain temperature and swaps per generation parameters. Eight chains were sampled every 4000 generations for a total of 10000 trees each, sampled from the posterior distribution. Those trees sampled prior the analysis reaching an average standard deviation of split frequencies of 0.02 were discarded as the burn-in, while the remaining trees were used to calculate the posterior probabilities of the individual clades. Default settings were used in MrBayes to set unconstrained branch lengths and uninformative topology priors.

Table S1. Sequences included in Mycenaceae phylogenetic analyses (Fig. 5) and corresponding GenBank accession numbers.

Species	Collection ID	Genbank Acc. Numbers	
		LSU	ITS
Campanophyllum proboscideum	TENN56402	AY230866	AY230866
Cheimonophyllum candidissimum	AFTOL 1765	DQ457654	DQ486687
Chondrostereum purpureum	CBS 352.53	MH868776	MH857243
Cruentomycena allochroa	HMJAU48199 (Holotype)	MW222659	MW222648

Cruentomycena allochroa	HMJAU48195	MW222660	MW222649
Cruentomycena kedrovayae	TENN60716	EU532597	EU517514
Cruentomycena orientalis	HMJAU47775	–	MW222646
Cruentomycena orientalis	HMJAU47726	–	MW222647
Cruentomycena viscidocruenta	K(M)117458	EU532598	EU517518
Crustomyces albidus	BJFC 033109 (Holotype)	NG_149056	NR_184516
Cyphella digitalis	AFTOL 663	AY635771	DQ486698
Cystostereum submurrayi	He 4379	ON117177	ON117192
Dictyopanus pusillus	8265	AY014291	AF289061
Effusomyces thailandicus	BJFC 023496 (Holotype)	NG_149059	NR_184518
Favolaschia andina	PMA<PAN>:KG0025	HM246679	HM246678
Favolaschia bannaensis	Dai 22587	ON870473	ON870497
Favolaschia bannaensis	Dai 22589	ON870474	ON870498
Favolaschia bannaensis	Dai 22590 (Holotype)	ON870475	ON870499
Favolaschia brevistipata	Dai 19855	MZ661743	MZ661773
Favolaschia brevistipata	Dai 19856	MZ661744	MZ661774
Favolaschia brevistipata	Dai 19780 (Holotype)	NG_088284	NR_177619
Favolaschia calocera	Dai 18663	MT293228	MT292322
Favolaschia crassispora	Dai 19871 (Holotype)	NG_241999	ON870501
Favolaschia crassispora	Dai 19769	ON870476	ON870500
Favolaschia flabelliformis	Dai 20010 (Holotype)	NG_242000	ON870502
Favolaschia flabelliformis	Dai 24354	OR260441	OR271272
Favolaschia longistipata	Dai 19799 (Holotype)	MZ661739	MZ661784
Favolaschia macropora	PMA<PAN>:KG0027 (Holo.)	HM246682	NR_132845
Favolaschia minutissima	Dai 20085	MZ661736	MZ661791
Favolaschia minutissima	Dai 20086 (Holotype)	NG_088283	NR_184479
Favolaschia peziziformis	ICMP15757	–	DQ026255
Favolaschia pustulosa	Dai 19758	MT293226	MT292325
Favolaschia pustulosa	Dai 19892	MT293227	MT292326
Favolaschia rigida	Dai 18566A (Holotype)	NG_242001	NR_189885
Favolaschia rigida	Dai 20764	ON870480	ON870505
Favolaschia semicircularis	Dai 19725 (Paratype)	ON870481	ON870507
Favolaschia semicircularis	Dai 19936 (Paratype)	ON870483	ON870509
Favolaschia semicircularis	Dai 19980 (Paratype)	ON870484	ON870511
Favolaschia semicircularis	Dai 19981 (Paratype)	ON870485	ON870512
Favolaschia semicircularis	Dai 22290 9Paratype)	ON870486	ON870513
Favolaschia semicircularis	Dai 22291 (Paratype)	ON870487	ON870514
Favolaschia semicircularis	Dai 22298 (Patratype)	ON870488	ON870515
Favolaschia subpustulosa	Dai 20719 (Holotype)	NG_242003	ON870518
Favolaschia tenuissima	Dai 22071 (Holotype)	NG_242004	ON870519

Favolaschia tenuissima	Dai 22072 (Paratype)	ON870491	ON870520
Favolaschia tephroleuca	Dai 22288 (Holotype)	NG_242005	ON870522
Favolaschia tephroleuca	Dai 22282 (Paratype)	ON870492	ON870521
Favolaschia tonkinensis	Dai 21955	ON870494	ON870523
Favolaschia tonkinensis	Dai 21956	ON870495	ON870524
Favolaschia tonkinensis	Dai 21965	ON870496	ON870526
Favolaschia tonkinensis	Dai 19704	OR260446	OR271277
Favolaschia xtbgensis	HKAS 121667 (Holotype)	OL413035	OL413036
Favolaschia xtbgensis	HKAS 121975	OL413044	OL413048
Favolashia claudopus	Dai 18663	MZ661734	MZ661776
Favolashia claudopus	Dai 18656 (Holotype)	MZ661735	MZ661775
Filoboletus keralensis	KFRIMH 1503 (Holotype)	OP429632	OP429631
Filoboletus manipularis	PL110619	MZ827877	MZ828043
Filoboletus pallescens	DED 8303	MH385336	MH414562
Filoboletus pallescens	BAP 654	MH385337	MH414563
Gloeostereum incarnatum	BCC 41461	KY614002	KY614001
Mycena abramsii	HMJAU43282	MK629348	MH396626
Mycena abramsii	HMJAU43523	MK629350	MH396628
Mycena adscendens	Aronsen120803	KT900140	KT900140
Mycena adscendens	Aronsen061119	KT900142	KT900142
Mycena aetites	CBS 222.47	MH867753	MH856226
Mycena aetities	CBS 221.47	MH867752	MH856225
Mycena aff. discobasis	BAP 658	MH385330	MH414554
Mycena aff. discobasis	DED 8211	MH385331	MH414555
Mycena aff. murina	UBC F14062	AF335444	AF335444
Mycena aff. pura	AFTOL 1486	DQ457688	DQ490643
Mycena aff. pura	TL9433	FN394647	FN394622
Mycena albiceps	SAT1518708	MF797659	KY777372
Mycena albiceps	MGW1504	MF797661	KY744173
Mycena algeriensis	HMJAU43798	MK722347	MK733295
Mycena alphitophora	BAP 591	MH385329	MH414553
Mycena amicta	AFTOL 1908	DQ457692	DQ490645
Mycena amicta	CBS 352.50	MH868170	MH856655
Mycena amicta	CBS 257.53	MH868722	MH857184
Mycena antennae	BAP 660 (Holotype)	MH385326	MH414550
Mycena arcangeliana	DM1051	MT644906	MT644906
Mycena asterina	DED 7812	PP795282	–
Mycena aurantiomarginata	CBS 357.50	MH868173	MH856657
Mycena bicystidiata	HMJAU43593	MK629354	MK309775
Mycena bicystidiata	HMJAU43648 (Holotype)	MK629359	NR_173285

Mycena breviseta	BAP 633	MH385327	MH414551
Mycena brunneoviolacea	BAP 656	MH385323	MH414546
Mycena bulliformis	BAP 547	KX513848	KX513844
Mycena caeruleomarginata	FFAAS:0357 (Holotype)	NG_153993	NR_182966
Mycena cahaya	ACL 134 (Holotype)	NG_060307	NR_154168
Mycena chlorophos	305759	AB512399	AB512312
Mycena chlorophos	ACL 145	KJ206953	KJ206974
Mycena citricolor	CBS 193.57	MH869233	MH857694
Mycena citrinomarginata	CBS 276.53	MH868733	MH857195
Mycena citrinomarginata	CBS 355.50	MH868171	MH856656
Mycena clavicularis	HMJAU43611	ON791547	ON791480
Mycena confinationis	PAMP:fungi-41 (Holotype)	NG_153925	MT764847
Mycena cristinae	JS347 (Holotype)	MT921384	MT921381
Mycena crocata	DM1009	MT644938	MT644938
Mycena deeptha	DM334g	–	JX481737
Mycena deeptha	CT16061502	–	MH400937
Mycena deformis	DED 8743	–	KX010907
Mycena diosma	STU:LK1191/2002	FN394644	FN394619
Mycena diosma	CBH400	FN394646	FN394617
Mycena discobasis	Stevani 08.04.05	PP795283	–
Mycena discogena	BAP 649	MH385332	MH414556
Mycena epipterygia	UBC F16307	EU486451	EU486451
Mycena epipterygia	UBC F19724	HQ604771	HQ604771
Mycena epipterygia	DED 8176	KX513846	KX513842
Mycena fera	PR-6462	PP795284	–
Mycena filopes	HMJAU43562	MK722350	MH396635
Mycena filopes	NN198	OP605599	OP604441
Mycena flos-nivum	CBS 364.50	MH868178	MH856661
Mycena flos-nivum	CBS 260.53	MH868724	MH857186
Mycena galericulata	AFTOL 727	AY647216	DQ404392
Mycena galericulata	TENN-F-014675h1	MN088380	MN088380
Mycena galericulata	TFB14649	MN088384	MN088384
Mycena galopus	UBC F19685	HM240534	HM240534
Mycena galopus	BAP 593	MH385324	MH414548
Mycena galopus	DM1023	MT644928	MT644928
Mycena globulispora	DED 8741	–	KX010908
Mycena globulispora	ACP1765	–	MG926696
Mycena gombakensis	LE-BIN 3510	–	OQ023329
Mycena griseotincta	HMJAU43800 (Holotype)	MK629363	MK309783
Mycena guzmanii	KUBOT-KRMK-2020-107	MW493147	MW493148

Mycena haematopus	UBC F19712	HQ604768	HQ604768
Mycena haematopus	ZRL20151471	KY418869	LT716053
Mycena haematopus	HMJAU43494	MK722351	MK733296
Mycena heteracantha	HMJAU43709	MK629362	MK309785
Mycena hygrophoroides	HMJAU43417 (Holotype)	MK629349	MK309780
Mycena illuminans	ACL 161	JX975217	KJ206975
Mycena illuminans	ACL 175	JX975218	KJ206976
Mycena illuminans	ACL 212	JX975219	KJ206980
Mycena inclinata	DM1052	MT644905	MT644905
Mycena inclinata	DM1035	MT644919	MT644919
Mycena indigotica	WEI 16-475 (Holotype)	MF993024	MF993026
Mycena jingyinga	KUC20220626_14	–	OR600259
Mycena jingyinga	KUC20220626_13	–	OR600258
Mycena jingyinga	LE-BIN 4556	–	OP997504
Mycena jingyinga	KUC20220626_09	–	OR600257
Mycena juniperifoliae	PAMP:fungi-40 (Holotype)	NG_153924	NR_173947
Mycena kentingensis	YSH-2014 (Holotype)	–	KC507796
Mycena lacrimans	RBNeto 25	PP795285	–
Mycena lasiopus	BAP 603	MH385333	MH414558
Mycena lazulina	WEI 18-367	MW540883	MW540882
Mycena leptocepahala	UBC F19740	HQ604773	HQ604773
Mycena leptocepahala	DM1046	MT644911	MT644911
Mycena longinqua	BAP 648 (Holotype)	MH385328	MH414552
Mycena luguensis	CT20160222 (Holotype)	–	MG324366
Mycena lumina	ACP1679 (Holotype)	–	NR_163298
Mycena lumina	ACP1720	–	MG926684
Mycena lux-coeli	Otsuki s.n.	PP795286	–
Mycena luxaeterna	DED 8087 (Holotype)	PP795287	–
Mycena luxfoliicola	ACP1791 (Holotype)	–	MG926688
Mycena luxfoliicola	ACP1684	–	MG926686
Mycena luxperpetua	PR-6463 (Holotype)	PP795288	–
Mycena maculata	CBS 235.47	MH867759	MH856231
Mycena maculata	CBS 237.47	MH867761	MH856232
Mycena maculata	CBS 238.47	MH867762	MH856233
Mycena margarita	BZ-4303	PP795289	PP789255
Mycena margarita	PR-5447	–	PP789256
Mycena miscanthi	HMJAU43573	MK629352	MK309777
Mycena miscanthi	HMJAU43584 (Holotype)	MK629353	MK309779
Mycena monticola	OSC 139197	JX287507	JX310425
Mycena mucor	AAronsen5-140914	–	KU861565

Mycena mucor	AAronsen7-051113	–	KU861566
Mycena nebula	ACP1659 (Holotype)	–	MG926685
Mycena nocticaelum	ACL258 (Holotype)	–	NR_156274
Mycena nocticaelum	ACL272	–	KJ206987
Mycena noctilucens	ACL 054	KJ206947	KJ206966
Mycena oboensis	BAP 669 (Holotype)	MH385334	MH414559
Mycena oculisnymphae	DED 8742(Holotype)	–	KX010909
Mycena oculisnymphae	DED 8734	–	KX010910
Mycena oculisnymphae	DUDA032	–	MT991424
Mycena oculisnymphae	DUDA085	–	MT991429
Mycena olivaceomarginata	CBS 227.47	MH867755	MH856227
Mycena olivaceomarginata	CBS 228.47	MH867756	MH856228
Mycena pasvikensis	AAronsen45-13	KU861557	KU861557
Mycena pasvikensis	AAronsen50-13	KU861558	KU861558
Mycena pearsoniana	HMJAU43826	MK722356	MK733305
Mycena perlae	ACP1669	–	MG926691
Mycena perlae	ACP1353 (Holotype)	–	MG926690
Mycena phaeonox	BAP 615 (Holotype)	MH385338	MH414564
Mycena plumbea	AFTOL 1631	DQ470813	DQ494677
Mycena polygramma	CBS 241.47	MH867765	MH856236
Mycena polygramma	CBS 243.47	MH867767	MH856238
Mycena pura	CBH358	FN394629	FN394608
Mycena pura	CBH371	FN394630	KF913023
Mycena pura	CBH367	FN394631	KF913022
Mycena pura f. alba	CBH410	FN394640	FN394595
Mycena pura f. lutea	DB2005/152	FN394635	FN394603
Mycena purpureofusca	UBC F19731	HQ604765	HQ604765
Mycena purpureofusca	UBC F19748	HQ604766	HQ604766
Mycena renati	CBS 358.50	MH868174	MH856658
Mycena rosea	CBH097	FN394628	FN394556
Mycena rosea	UP2	FN394632	FN394550
Mycena rosea	TL12393	FN394641	FN394555
Mycena rosea	F784	OR602444	OR625707
Mycena rubromarginata	TL12780	KX513849	KX513845
Mycena rubromarginata	CBS 268.48	MH867891	MH856338
Mycena sanguinolenta	430360	AB512401	AB512311
Mycena sanguinolenta	KUBOT-KRMK-2020-57	MW446189	MW446185
Mycena seminau	ACL 136 (Holotype)	KJ206952	KF537250
Mycena seminau	ACL 308	KJ206964	KF537252
Mycena semivestipes	HMJAU43825	MK722358	MK733308

Mycena silvae-nigrae	HMJAU43815	MK722359	MK733310
Mycena silvaelucens	BAP 568 (Holotype)	PP795290	–
Mycena sinar	ACL 092	KJ206948	KF537247
Mycena sinar	ACL 135 (Holotype)	NG_069189	NR_154169
Mycena sinar v. tangkaisinar	ACL 307 (Holotype)	NR_154171	NR_154171
Mycena singeri	PR-6456	PP795291	–
Mycena solis	BAP 592 (Holotype)	MH385325	MH414549
Mycena stylobates	MCVE 20013	PP795292	–
Mycena tenerrima	UBC:F19725	HQ604774	HQ604774
Mycena tenerrima	G.M. 2014-09-30.5	MZ467320	MZ467320
Mycena tenuicorticola	RIKI-2017120301	MT764851	MT764848
Mycena vulgaris	CBS 248.47	MH867770	MH856240
Mycena xantholeuca	CBS 370.50	MH868180	MH856663
Mycena xantholeuca	CBS 371.50	MH868181	MH856664
Mycena zephirus	CBS 272.48	MH867893	MH856340
Mycena zephirus	CBS 273.48	MH867894	MH856341
Panellus bambusicola	Dai 19895	MT396582	MT363742
Panellus bambusicola	Dai 19896	MT396583	MT363743
Panellus bambusicola	Dai 19897	MT396584	MT363744
Panellus longistipatus	Dai 22487 (Holotype)	NG_088355	NR_182993
Panellus longistipatus	Dai 22065	ON074731	ON074667
Panellus luminescens	ACL205	KJ206955	KJ206979
Panellus luxfilamentus	ACL274 (Holotype)	KJ206959	KJ206988
Panellus minutissimus	Dai 22052 (Holotype)	ON074733	ON074669
Panellus minutissimus	Dai 22068	ON074734	ON074670
Panellus palmicola	Dai 19719	ON074736	ON074672
Panellus palmicola	Dai 22329	ON074737	ON074673
Panellus palmicola	Dai 22334	ON074739	ON074678
Panellus pusillus	KUBOT-KRMK-2020-145	MW555775	MW555779
Panellus stipticus	CBS 332.34	MH867060	MH855555
Panellus stipticus	CBS 333.34	MH867061	MH855556
Panellus stipticus	CBS 335.34	MH867062	MH855557
Panellus yunnanensis	Dai 20728	MT300511	MT300504
Panellus yunnanensis	Dai 20729	MT300512	MT300505
Panellus yunnanensis	Dai 20730 (Holotype)	MT300513	MT300506
Parvodontia austrosinensis	BJFC 024249 (Holotype)	NG_149060	NR_184519
Resinomycena montana	SR 7301	ON332045	ON332045
Resinomycena montana	SR 7374	ON332046	ON332046
Resinomycena petarensis	DED 8736	–	KX010911
Resinomycena petarensis	DED 8695 (Holotype)	–	NR_160456

Resinomycena rhododendri	TENN50793	EU532599	EU517509
Resinomycena sp.	NN137	ON775502	ON775523
Resinomycena sp.	HMJAU47840	MW222663	MW222653
Roridomyces apendiculatus	NY153	ON775510	ON775531
Roridomyces glutinosus	HUIF50300	ON775508	ON775529
Roridomyces lamprosporus	BAP 634	MH385339	–
Roridomyces phyllostachydis	MFLU19-2825 (Holotype)	MT275657	MT274525
Roridomyces phyllostachydis	MFLU19-2826	MT275658	MT274526
Roridomyces praeclarus	NY0871	ON775509	ON775530
Roridomyces roridus	NOFQB24_Q35	LC757539	LC757538
Roridomyces roridus	NOFFB5_F8	LC757620	LC757619
Roridomyces roridus	NN226	ON775506	ON775527
Roridomyces sp.	HMJAU48013	MW222658	MW222642
Roridomyces sp.	SG111	MW226886	MW226885
Roridomyces sp.	NN160	ON775503	ON775524
Roridomyces sp.	NN105	ON775504	ON775525
Roridomyces sp.	NN235	ON775507	ON775528
Roridomyces sp.	NN162z	ON775511	ON775532
Roridomyces sp.	NN17	ON775513	ON775534
Roridomyces sp.	NN19	ON775514	ON775535
Roridomyces sp.	NN225	ON775515	ON775538
